# Relationship between Locomotive Syndrome and Cortical Bone Thickness and Trabecular Bone Density in a Community-dwelling Postmenopausal Population

**DOI:** 10.1298/ptr.25-E10368

**Published:** 2025-11-15

**Authors:** Satoko NAKANO, Etsuko OZAKI, Wataru NAKANO, Michitaka KATO, Yasunari KURITA, Daisuke TAKAGI, Daisuke MATSUI, Naoyuki TAKASHIMA

**Affiliations:** 1 Department of Shizuoka Physical Therapy, Faculty of Health Science, Tokoha University, Japan; 2 Department of Epidemiology for Community Health and Medicine, Kyoto Prefectural University of Medicine Graduate School of Medical Science, Japan; 3 Department of Nursing, Faculty of Health and Medical Sciences, Kyoto University of Advanced Science, Japan; 4 Department of Health Sciences, Division of Rehabilitation Sciences, Nagoya City University of Medicine, Japan

**Keywords:** Postmenopausal women, Locomotive syndrome, Cortical bone thickness, Trabecular bone density, Bone strength

## Abstract

**Objectives:**

We investigated the association between locomotive syndrome (LS), cortical bone thickness, and trabecular bone density in community-dwelling postmenopausal women.

**Methods:**

In total, 1405 postmenopausal women aged ≥50 years were analyzed from the Japan Multi-Institutional Collaborative Cohort (J-MICC) Study. LS was assessed using the stand-up test, 2-step test, and the 25-question Geriatric Locomotive Function Scale. The cortical bone thickness and trabecular bone density at the radius were assessed using a quantitative ultrasound device capable of distinguishing between the cortical and trabecular components. Demographic and bone-related variables were compared between participants with and without LS using the t-test and chi-squared test. Multivariate linear regression analyses were performed with cortical bone thickness or trabecular bone density as the dependent variable and LS status as the independent variable, after adjusting for age and lifestyle factors.

**Results:**

Of all participants, 892 (63.5%) had LS. Multiple linear regression analysis showed that LS was independently associated with reduced cortical bone thickness (β = −0.076, p = 0.001) and trabecular bone density (β = −0.109, p <0.001). This association was statistically significant in both age groups (<65 and ≥65 years).

**Conclusions:**

In postmenopausal women, LS was independently associated with cortical bone thinning and trabecular bone loss. Our findings suggest that bone quality may play an important role in the prevention and management of LS, indicating that future strategies should consider both bone density and bone quality.

## Introduction

With Japan’s aging population, extending healthy life expectancy through preventive measures and improving medical care have become urgent issues. Locomotive syndrome (LS), proposed by the Japanese Orthopedic Association (JOA), is defined as a condition in which mobility is impaired due to musculoskeletal disorders, with an increased risk of requiring nursing care or becoming bedridden^1)^. The prevalence of LS is higher in women than in men, with approximately 40% of women in their 40s being affected, indicating that LS often develops in the early stages of middle age^2)^. Osteoporosis has been reported as a primary comorbidity of LS^3,4)^, and the relationship between LS and bone strength appears bidirectional. Consequently, the current osteoporosis guidelines recommend concurrent prevention strategies targeting both LS and osteoporosis^5)^. Particularly in women, preventing LS may reduce fall-related fractures and the subsequent transition to dependence or care.

Bone strength is determined by 2 major components: bone mineral density (BMD) and bone quality, with approximately 70% attributable to BMD and 30% to bone quality^6)^. While BMD has traditionally served as a surrogate marker of bone strength, bone quality also plays a crucial role. Bone quality includes characteristics such as geometry, microarchitecture, remodeling rate, accumulation of microdamage, and degree of mineralization^7)^. Among these, cortical bone properties, particularly cortical thickness, have been recognized as significant contributors to bone strength, especially in older adults^8,9)^.

Chronic inflammation and oxidative stress resulting from lifestyle-related diseases can degrade bone quality, independent of bone density, thereby increasing the risk of fragility fractures^6,7,10)^. In contrast, resistance exercises and weight-bearing activities have been shown to improve bone strength by promoting periosteal apposition and increasing cortical bone thickness, in addition to increasing BMD^11,12)^. Therefore, chronic inflammation and oxidative stress associated with lifestyle-related diseases^10,13–16)^, as well as reduced mechanical loading due to muscle weakness^17,18)^ and decreased physical activity^19)^, may contribute not only to a decline in BMD but also to cortical bone thinning. Accordingly, when evaluating the association between bone strength and LS, it is essential to appropriately adjust for lifestyle-related and disease-associated factors as confounding variables. However, previous studies^20,21)^ examining the relationship between LS and bone strength have generally been limited by small sample sizes and have primarily focused on associations with trabecular bone density. To our knowledge, no large-scale, community-based investigation has yet examined this association in detail.

Therefore, we aimed to examine the relationship between the LS and cortical bone thickness in postmenopausal women participating in a regional community-based cohort study, using a quantitative ultrasound device capable of separately assessing the trabecular and cortical bones. By elucidating this relationship, physical therapists may obtain an evidence base for incorporating strength training and aerobic exercise into clinical practice as strategies to maintain bone health and to aid in the prevention and management of LS.

## Methods

### Study participants

**Fig. 1.**
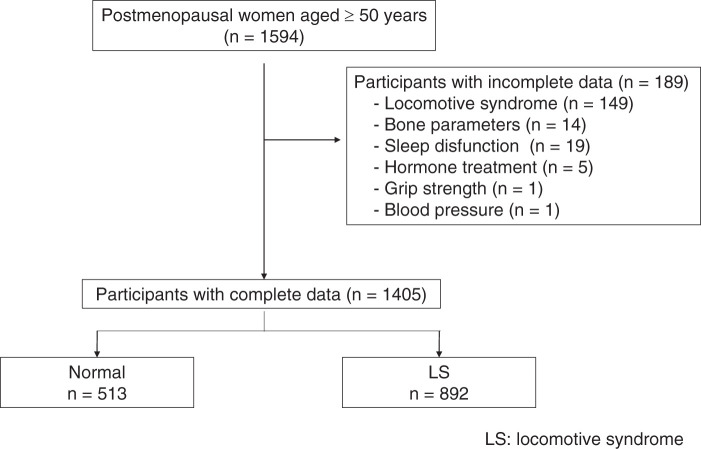
Flow diagram of participant selection. Among 1594 postmenopausal women aged ≥50 years, 189 were excluded due to incomplete data on locomotive syndrome (n = 149), bone parameters (n = 14), sleep dysfunction (n = 19), hormone treatment (n = 5), grip strength (n = 1), or blood pressure (n = 1). The final analysis included 1405 participants, who were classified into normal (n = 513) and LS (n = 892) groups. LS, locomotive syndrome

From November 2013 to December 2017, 1594 postmenopausal women aged ≥50 years living in the Kyoto area participated in the Japan Multi-Institutional Collaborative Cohort (J-MICC) Study^22,23)^. Of these, 1405 with complete data were included in the analysis (Fig. 1). Written informed consent was obtained from all participants before their participation in the study. Ethical approval was obtained from the Institutional Ethics Committee of the Kyoto Prefectural University of Medicine (ERB-E-36). This study was conducted in accordance with the principles of the World Medical Association’s Declaration of Helsinki.

### Assessment of cortical bone thickness and trabecular bone density

Cortical bone thickness and trabecular bone density were measured in the radius of the non-dominant arm using an ultrasonic bone densitometer (LD-100; Oyo Electric, Kyoto, Japan). The device calculates bone parameters such as cortical bone thickness (mm) and trabecular bone density (mg/cm^3^) using the propagation speed (m/s) and attenuation (dB) of both fast and slow waves. The validity of the LD-100 has previously been reported^24–26)^.

### Assessment of LS

According to the Japanese Orthopaedic Association (JOA)’s proposal^27)^, 3 tests were used: the 2-step test, stand-up test, and 25-question Geriatric Locomotive Function Scale (GLFS-25). The overall LS status of each participant was determined by combining the results of the 3 tests. If any one of the 3 tests was positive, the participant was classified as “with LS”; otherwise, they were classified as “without LS”.

### 2-Step test

Starting from an upright standing posture, the participants took a maximum of 2 steps forward without losing balance, swinging their arms, or using rebound. The lengths of these 2 steps were measured. The 2-step test was conducted twice to obtain optimal results, and the higher value was used for the analysis. The 2-step test score was calculated using the following formula: length of the 2 steps (cm) divided by height (cm). A 2-step value of <1.3 was considered LS-positive^27)^.

### Stand-up test

The participants rose from 4 different chairs (10, 20, 30, and 40 cm) using either one or both legs. They were instructed not to swing their arms or use rebound and to maintain the standing position for at least 3 seconds. Failure to stand from a height of 40 cm with both legs or one leg was considered LS-positive^27)^.

### 25-question GLFS

The GLFS-25 is a self-administered questionnaire consisting of 25 items: 4 on pain, 16 on activities of daily living, 3 on social functioning, and 2 on mental health^28)^. Each item is rated on an ordinal scale ranging from 0 to 4, with higher scores indicating more severe symptoms. GLFS-25 scores ≥7 were considered LS-positive.

### Assessment of muscle strength

Both left and right grip strengths were measured once using a Smedley hand dynamometer (Matsumiya Medical Precision Machinery, Tokyo, Japan). Measurements were taken in the standing position, with the shoulder in a neutral position and the elbow fully extended. The higher of the 2 values was used for the analysis.

### Assessment of covariates

Blood tests and blood pressure measurements were routinely performed, and a self-administered questionnaire was used to collect data on the menopause status, history of hormone treatment, smoking status, alcohol consumption, medication use, sleep disorders, and physical activity. Blood samples were collected in the fasting state after at least 6 h of overnight fasting. Blood pressure was measured manually.

Based on the questionnaire data, menopause status and history of hormone treatment were confirmed, and participants who currently smoked or consumed alcohol were defined as smokers or alcohol consumers. Hypertension was defined as the use of antihypertensive drugs, systolic blood pressure ≥140 mmHg, or diastolic blood pressure ≥90 mmHg. Diabetes mellitus was defined as the use of oral hypoglycemic drugs or a fasting blood sugar level ≥126 mg/dL. Dyslipidemia was defined as taking lipid-lowering drugs, triglycerides ≥150 mg/dL, low-density lipoprotein cholesterol ≥140 mg/dL, or high-density lipoprotein cholesterol <40 mg/dL, or non-high-density lipoprotein cholesterol ≥170 mg/dL. Renal dysfunction was defined as an estimated glomerular filtration rate of <90 mL/min/1.73 m^2^. Sleep quality was assessed using the Pittsburgh Sleep Questionnaire^29)^, and a score ≥5 was defined as a sleep disorder^30)^. Physical activity was measured using a self-administered questionnaire that collected information on the type, frequency, and duration of exercise and was calculated as metabolic equivalents (METs) per week^31)^.

### Statistical analysis

Group comparisons between participants with and without LS for demographic and bone-related variables were conducted using t-tests for continuous variables and chi-squared tests for categorical variables. Subgroup analyses by age (<65 years vs. ≥65 years) were also performed. Multiple linear regression analyses were conducted with cortical bone thickness or trabecular bone density as the dependent variable and LS status as the independent variable. Covariates included age, body mass index (BMI), history of hormone treatment, smoking, alcohol consumption, hypertension, diabetes, dyslipidemia, renal dysfunction, sleep disorders, level of physical activity (METs), and grip strength. These covariates were selected because they are known to be associated with both LS and bone health, based on previous studies, and were therefore considered potential confounding factors^2,10)^. The models were similarly adjusted and stratified by age group (<65 vs. ≥65 years) to assess age-specific associations between LS and bone strength. All analyses were conducted at a significance level of p <0.05 using SPSS version 25.0 (IBM, Armonk, NY, USA).

## Results

In total, 1594 postmenopausal women aged ≥50 years were enrolled in this study. Participants with incomplete data were excluded (n = 189), including those with missing information on LS (n = 149), bone parameters (n = 14), sleep dysfunction (n = 19), hormone treatment (n = 5), grip strength (n = 1), and blood pressure (n = 1). Consequently, complete data were available for 1405 participants (Fig. 1). Among them, 892 (63.5%) had LS (Table 1).

**Table 1.** Clinical characteristics of the study participants

	Total (n = 1405)
Age, years	63.1 ± 6.7
BMI, kg/m^2^	21.6 ± 3.1
Hormone treatment, n (%)	223 (15.9)
Smoking, n (%)	42 (3.0)
Alcohol consumption, n (%)	669 (47.6)
Hypertension, n (%)	537 (38.2)
Diabetes mellitus, n (%)	52 (3.7)
Dyslipidemia, n (%)	634 (45.1)
Renal dysfunction, n (%)	252 (17.9)
Sleep disorder, n (%)	606 (43.1)
Physical activity, METs-h/week	2.8 ± 3.5
Grip strength, kg	29.1 ± 4.9
Locomotive syndrome	
With, n (%)	892 (63.5)
Bone parameters	
Cortical bone thickness, mm	2.8 ± 0.7
Cortical bone thickness, %YAM	72.5 ± 18.9
Trabecular bone density, mg/cm^3^	153.4 ± 47.3
Trabecular bone density, %YAM	75.1 ± 23.1

Values are mean ± standard deviation or frequency and percentage.

The percentages of cortical bone thickness and trabecular bone density relative to the young adult mean (%YAM) represent the ratio of each parameter to the average values observed in healthy young adults (aged 20–44 years).

BMI, body mass index; MET, metabolic equivalent; h, hours; %YAM, %young adult mean

Compared to the without LS group, the with LS group was significantly older, had a higher BMI, and a higher prevalence of hypertension and sleep disorders, and grip strength was significantly reduced. The with LS group also showed significantly reduced cortical bone thickness and trabecular bone density compared to the without LS group. These differences persisted across the age groups (Table 2).

**Table 2.** Clinical and bone characteristics by age group and locomotive syndrome status

	Total		p-Value	<65 years		p-Value	≥65 years		p-Value
	Without LS (n = 513)	With LS (n = 892)		Without LS (n = 340)	With LS (n = 408)		Without LS (n = 173)	With LS (n = 484)	
Age, years	61.2 ± 6.2	64.2 ± 6.7	<0.001	57.5 ± 4.1	58.0 ± 3.9	0.173	68.4 ± 2.4	69.5 ± 2.8	<0.001
BMI, kg/m^2^	21.1 ± 2.7	21.8 ± 3.2	<0.001	20.9 ± 2.7	21.7 ± 3.4	<0.001	21.4 ± 2.8	22.0 ± 3.1	0.023
Hormone treatment, n (%)	86 (16.8)	137 (15.4)	0.488	56 (16.5)	74 (18.1)	0.549	30 (17.3)	63 (13.0)	0.161
Smoking, n (%)	15 (2.9)	27 (3.0)	0.913	13 (3.8)	13 (3.2)	0.636	2 (1.2)	14 (2.9)	0.203
Alcohol consumption, n (%)	259 (50.5)	410 (46.0)	0.102	182 (53.5)	216 (52.9)	0.872	77 (44.5)	194 (40.1)	0.310
Hypertension, n (%)	165 (32.2)	372 (41.7)	<0.001	85 (25.0)	124 (30.4)	0.102	80 (46.2)	248 (51.2)	0.259
Diabetes mellitus, n (%)	13 (2.5)	39 (4.4)	0.079	5 (1.5)	9 (2.2)	0.460	8 (4.6)	30 (6.2)	0.447
Dyslipidemia, n (%)	216 (42.1)	418 (46.9)	0.085	139 (40.9)	192 (47.1)	0.090	77 (44.5)	226 (46.7)	0.621
Renal dysfunction, n (%)	96 (18.7)	156 (17.5)	0.565	51 (15.0)	52 (12.7)	0.373	45 (26.0)	104 (21.5)	0.223
Sleep disorder, n (%)	187 (36.5)	419 (47.0)	<0.001	120 (35.3)	188 (46.1)	0.003	67 (38.7)	231 (47.7)	0.041
Physical activity, METs-h/week	2.9 ± 3.2	2.7 ± 3.6	0.273	2.5 ± 3.0	1.9 ± 2.4	0.006	3.7 ± 0.6	3.3 ± 4.1	0.320
Grip strength, kg	30.3 ± 4.6	28.4 ± 5.0	<0.001	30.9 ± 4.6	29.7 ± 4.8	<0.001	29.0 ± 4.2	27.3 ± 4.8	<0.001
Bone parameters									
Cortical bone thickness, mm	3.0 ± 0.7	2.7 ± 0.7	<0.001	3.2 ± 0.7	3.0 ± 0.7	<0.001	2.6 ± 0.7	2.5 ± 0.6	<0.001
Cortical bone thickness, %YAM	77.6 ± 18.7	69.5 ± 18.3	<0.001	82.6 ± 17.7	77.4 ± 17.2	<0.001	67.8 ± 16.8	62.8 ± 16.4	<0.001
Trabecular bone density, mg/cm^3^	163.5 ± 47.2	147.6 ± 46.4	<0.001	171.9 ± 47.8	164.5 ± 47.5	0.034	147.0 ± 41.2	133.4 ± 40.4	<0.001
Trabecular bone density, %YAM	80.1 ± 23.1	72.3 ± 22.7	<0.001	84.2 ± 23.4	80.6 ± 23.3	0.034	71.9 ± 20.0	65.4 ± 19.8	<0.001

Values are mean ± standard deviation or frequency and percentage.

The percentages of cortical bone thickness and trabecular bone density relative to the young adult mean (%YAM) represent the ratio of each parameter to the average values observed in healthy young adults (aged 20–44 years).

LS, locomotive syndrome; BMI, body mass index; h, hours; MET, metabolic equivalent; %YAM, %young adult mean

In the multiple linear regression analysis, the with LS group had a statistically significant and independent negative effect on both cortical bone thickness (β = −0.076, p = 0.001) and trabecular bone density (β = −0.109, p <0.001) (Table 3). These associations were statistically significant in both age groups (<65 and ≥65 years) (Table 4).

**Table 3.** Multiple regression analyses for the cortical bone thickness and trabecular bone density

	Cortical bone thickness			Trabecular bone density		
	β	95% CI	p-Value	β	95% CI	p-Value
Age, years	−0.413	−0.051, −0.040	<0.001	−0.363	−3.159, −2.409	<0.001
BMI, kg/m^2^	−0.209	−0.061, −0.039	<0.001	0.236	2.860, 4.393	<0.001
Hormone treatment, yes	−0.002	−0.093, 0.085	0.928	0.023	−3.073, 9.038	0.334
Smoking, yes	−0.007	−0.223, 0.160	0.750	−0.039	−23.904, 9.038	0.101
Alcohol consumption, yes	−0.021	−0.096, 0.035	0.358	0.007	−3.814, 5.097	0.778
Hypertension, yes	−0.006	−0.080, 0.063	0.810	0.015	−3.394, 6.311	0.555
Diabetes mellitus, yes	−0.029	−0.288, 0.061	0.201	0.001	−11.565, 12.128	0.963
Dyslipidemia, yes	0.002	−0.063, 0.069	0.940	−0.007	−5.143, 3.818	0.772
Renal dysfunction, yes	−0.012	−0.109, 0.063	0.604	0.023	−2.961, 8.743	0.333
Sleep disorder, yes	0.067	0.033, 0.165	0.003	0.017	−2.871, 6.110	0.479
Physical activity, METs-h/week	0.036	−0.002, 0.017	0.120	−0.002	−0.678, 0.615	0.924
Grip strength, kg	0.127	0.012, 0.026	<0.001	−0.011	−0.586, 0.379	0.674
With locomotive syndrome	−0.076	−0.186, −0.046	0.001	−0.109	−15.494, −5.948	<0.001

For categorical variables; “yes” indicates the presence of a condition or behavior. The reference group was “no”.

CI, confidence interval; BMI, body mass index; MET, metabolic equivalent

**Table 4.** Age-stratified sensitivity analyses of multiple regression models for bone parameters

	Cortical bone thickness			Trabecular bone density		
	β	95% CI	p-Value	β	95% CI	p-Value
Participants aged <65 years						
With locomotive syndrome	−0.090	−0.216, −0.033	0.008	−0.097	−15.903, −2.707	0.006
Participants aged ≥65 years						
With locomotive syndrome	−0.078	−0.225, −0.004	0.043	−0.140	−20.063, −6.007	<0.001

Adjusted for age, BMI, hormone treatment, smoking, alcohol consumption, hypertension, diabetes mellitus, dyslipidemia, renal dysfunction, sleep disorders, physical activity, and grip strength.

CI, confidence interval; BMI, body mass index; h, hours

## Discussion

This study investigated the associations among LS, cortical bone thickness, and trabecular bone density in 1405 postmenopausal women residing in the community. The results showed that both cortical bone thickness and trabecular bone density were significantly lower in the with LS group than in the without LS group. This association was consistently observed across age groups, and in the multivariate analysis, LS was an independent negative predictor of both cortical bone thickness and trabecular bone density. The novelty of this study lies in elucidating the association between LS, cortical bone thickness, and trabecular bone density in a large community-based sample of postmenopausal women.

In the present study, the prevalence of LS was 63.5% among community-dwelling postmenopausal women. This prevalence is comparable to previous epidemiological studies in Japan, including the Research on Osteoarthritis/Osteoporosis Against Disability study, which have reported LS prevalence rates ranging from approximately 50% to 70% in older adults^2,3)^. These findings suggest that the participants in our study shared a generally similar background with those of previous large-scale Japanese cohorts.

Cortical bone thickness is recognized as a key morphological determinant of bone strength, particularly in aging populations^32,33)^. Although it does not encompass all the aspects of bone quality, it serves as an accessible and clinically relevant marker of structural integrity. Cortical bone becomes thinner with age, and structural fragility is known to progress particularly after menopause due to increased bone resorption^7)^. To the best of our knowledge, no previous studies have reported an association between LS and cortical bone in postmenopausal women. The present findings are the first to demonstrate a significant association between LS and cortical bone thinning, suggesting that LS may influence not only bone density but also bone quality. Furthermore, the LS group had a higher prevalence of sleep disorders and a significantly lower grip strength. Since these factors were also significantly associated with cortical bone thickness, it is possible that, in addition to the direct association between LS and cortical bone thinning, reduced muscle strength and lifestyle deterioration associated with LS may further contribute to the decline in bone quality.

The present study also confirmed the association between LS and reduced trabecular bone density. Several previous studies have reported associations between osteoporosis and LS^18)^, as well as between bone density and LS^20,21)^. These findings are generally consistent with those of previous studies and further contribute to the field by utilizing a larger sample size and accounting for potential confounding factors such as diabetes^13)^ and sleep disorders^15)^, which are known risk factors for LS. These methodological features may have enhanced the reliability and applicability of this study’s conclusions.

The independent associations between LS and cortical and trabecular bone deterioration, together with the association between muscle strength and cortical bone thinning, suggest that preventing LS and improving muscle strength may be effective strategies for promoting bone health, particularly cortical bone integrity. Furthermore, the association between LS and bone health was observed in both age groups—those <65 years and those aged ≥65 years. However, the prevalence of LS was high in both groups, reaching 54.5% in individuals <65 years and 73.7% in those aged ≥65 years, indicating the necessity of earlier preventive interventions.

Exercise contributes to the maintenance of bone strength by stimulating osteoblastic activity and suppressing bone resorption^34,35)^. Notably, moderate- to high-intensity exercise has been shown to exert beneficial effects on both trabecular and cortical bone^12,36)^, whereas low-intensity exercise, such as walking, primarily affects the trabecular bone and has limited impact on the cortical bone^34)^. High-intensity physical activity has been suggested to play a key role in preventing LS and sarcopenia^37,38)^. Nevertheless, according to a national survey in Japan, only 49.4% of women reported engaging in regular physical activity. Among them, 60.5% reported walking, and only 10.8% engaged in strength training^39)^. Our findings may provide implications for physical therapy practice. Given that cortical bone thickness has been reported to be associated with muscle strength^40)^, incorporating resistance training into exercise instruction might be a promising approach for maintaining bone health. Such interventions could potentially contribute to the prevention and improvement of LS by addressing both muscle weakness and bone fragility.

This study has several limitations. First, since this study was conducted among volunteers, the participants were slightly younger and had stronger grip strength than those in a previously established large-scale population-based cohort (average age 65.6 years)^2)^, suggesting a potential selection bias. However, since a significant association between LS and bone strength was observed even in this relatively younger population, these findings indicate the importance of initiating preventive interventions for LS at an earlier stage rather than waiting until musculoskeletal decline becomes clinically evident. Additionally, bone strength was assessed using ultrasound rather than dual-energy X-ray absorptiometry, and although correlation with peripheral quantitative computed tomography has been reported, the impact of differences in assessment methods must be considered^41,42)^. Furthermore, the measurement site was the radius, which may have different functional correlations with the proximal femur and tibia, where fractures due to falls are common^40)^. Finally, this was a cross-sectional study; therefore, there are limitations in interpreting causal relationships. Future longitudinal studies are needed for further validation.

## Conclusions

This study demonstrated that LS was independently associated with decreased trabecular bone density and cortical bone thinning in community-dwelling postmenopausal women. Our findings suggest that bone quality may play an important role in the prevention and management of LS, indicating that future strategies should consider both bone density and bone quality.
